# Mechanical Properties of a Soy Protein Isolate–Grafted–Acrylate (SGA) Copolymer Used for Wood Coatings

**DOI:** 10.3390/polym12051137

**Published:** 2020-05-15

**Authors:** Bin Feng, Di Wang, Yuhui Li, Junpeng Qian, Chenlei Yu, Mingsi Wang, Danni Luo, Shuangying Wei

**Affiliations:** 1 College of Material Science and Engineering, Northeast Forestry University, Harbin 150040, China; nefufengbin@163.com (B.F.); liyuhuigege@126.com (Y.L.); qjp15940188486@163.com (J.Q.); yuchenlei925@163.com (C.Y.); wms991219@163.com (M.W.); n1256367845@163.com (D.L.); 2 Key Laboratory of Bio-Based Material Science and Technology, Northeast Forestry University, Harbin 150040, China

**Keywords:** soy protein isolate, polyacrylate, graft copolymerization, waterborne wood coating

## Abstract

Changing demands have led to rapidly growing interest in the modification of waterborne wood coatings. To improve the performance of a polyacrylate wood coating, especially the strength, hardness, and abrasion resistance of the film, a soy protein isolate–grafted–acrylate (SGA) copolymer was prepared in an aqueous solution with ammonium persulfate (APS) as an initiator and sodium pyrosulfite (SPS) as an unfolding agent for the soybean protein isolate (SPI). The emulsion was characterized using transmission electron microscopy, Fourier-transform infrared spectroscopy (FTIR), and a particle size analyzer. Furthermore, the mechanical properties of the film, including the tensile strength, elastic modulus, elongation at break, and pencil hardness, were measured. The results showed that the glass transition temperature of the polyacrylic resin decreased to 35 °C after the SPI grafting. The elastic modulus of the film increased from 0.317 to 46.949 MPa, and the elongation at break decreased from 453.133% to 187.125% as the addition of SPI varied from 0 to 4 g, respectively. The pencil hardness of the wood coating increased from HB to 3H. This paper proposes a feasible route for the utilization of SPI for wood coatings.

## 1. Introduction

In recent years, waterborne wood coatings have frequently been used in the decoration of homes, hotels, and wood furniture in China because they are non-toxic and environmentally friendly, and they release low amounts of volatile organic compounds (VOCs) [1,2]. Polyacrylate waterborne coatings are one of the main varieties of wood coatings with many advantages, such as exhibiting strong adhesion, forming colorless coatings, exhibiting high transparency, and possessing high solid contents. However, further applications of acrylate waterborne coatings are limited by their low hardness values and poor mechanical properties [3,4,5].

Various efforts have been made to improve the mechanical properties of polyacrylate waterborne coatings, such as copolymerization with polyurethane [6,7,8,9,10], silicone modification [11], treatment with nanoparticles [12,13,14], epoxy modification [15], microemulsion polymerization, microemulsion hybrid polymerization [16,17], and other methods [18,19]. The hardness values and mechanical properties of the obtained coatings were improved by these methods, but nonrenewable and nondegradable petrochemical-based materials were consumed. In the past few years, it has become a development trend to substitute petrochemical-based materials with biomass materials in the manufacture of wood coatings due to increased concern over environmental protection and stricter legislation [20,21,22,23,24,25,26,27,28].

The soy protein present in various soy products has received extensive attention from researchers due to its low cost, biodegradability, lack of health risks, and environmental friendliness [21,24]. Glycinin (11S globulin) and conglycinin (7S globulin) are two important constituents of soy protein, and they are mainly present in extracted soy protein [29,30]. Soybean protein isolate (SPI), which contains -NH_2_, -OH, -COOH, and -SH groups, contains the highest percentage of protein of all extracted soy protein forms (>90%) [31,32,33]. Pattanai and Sutar studied the graft copolymerization of polylactide onto SPI backbones by ring-opening polymerization in the presence of the Sn(Oct)_2_ catalyst [34]. SPI was graft copolymerized with styrene using ammonium cerium nitrate and potassium persulfate as the reaction initiator [35]. Yang et al. investigated the graft copolymerization of methyl methacrylate (MMA) onto SPI in an 8 mol/L urea aqueous solution with β-mercaptoethanol as an unfolding agent for SPI, a chain transfer agent, and ammonium persulfate (APS) as an initiator [36]. Gonzalez and Alvarez described SPI modification through a grafting reaction with MMA by replacing β-mercaptoethanol with sodium bisulfite as the protein unfolding agent [21]. In summary, many functional groups in SPI have been used for various applications.

**Figure 1:**
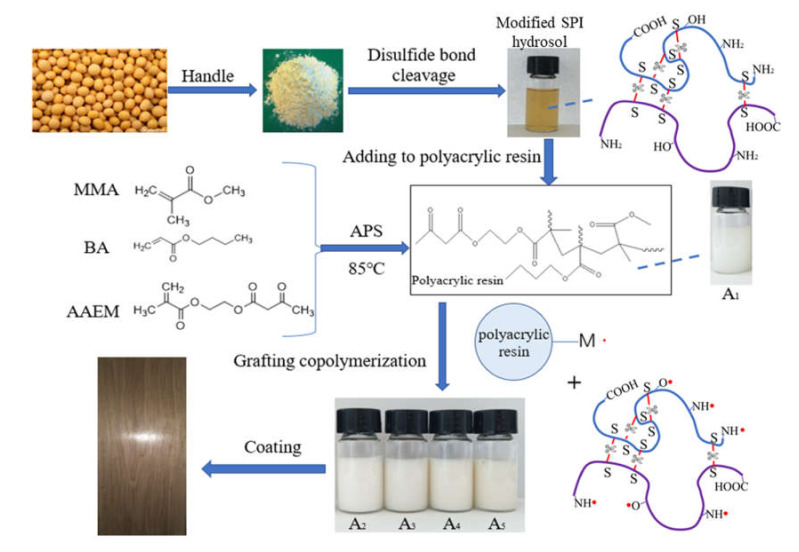
Synthesis of soybean protein isolate (SPI)-modified polyacrylate.

In this study, polyacrylate was modified by the grafting copolymerization of SPI biomass material. The soy protein isolate–grafted–acrylate (SGA) was applied for wood coating. The disulfide bond was cut by sodium metabisulfite to produce more amino, hydroxyl, and sulfur active sites under high-temperature and alkaline conditions. The amino and hydroxyl groups were attacked by an initiator, ammonium persulfate, to form radicals, and graft copolymerization with the acrylate monomer occurred [24,37,38,39]. In this way, more cross-linking sites were added to the system to increase the cross-linking density. The purpose of this study was to improve the mechanical performance and hardness of the waterborne polyacrylate wood coating. The synthesis route is shown in Figure 1.

## 2. Experimental

### 2.1. Materials

The soy protein isolate was obtained from Shansong Biological Products Co., Ltd (Linyi, China) (Protein(%) ≥90). The methyl methacrylate (MMA), n-butyl acrylate (BA), ammonium persulfate (APS), sodium dodecyl sulfate (SDS), and sodium hydroxide were obtained from Shanghai Macklin (China). The 2-(methacryloyloxy)ethyl acetoacetate (AAEM), sodium pyrosulfite (SPS), and OP-10 emulsifier were obtained from Shanghai Aladdin (Shanghai, China).

### 2.2. Methods

#### 2.2.1. Modification of Soy Protein Isolate

First, 1, 3, 4, and 6 g of soy protein isolate were mixed with 50 mL of an alkaline solution containing 0.2% NaOH by mass, followed by stirring at 95 °C for 60 min to prepare an SPI alkaline suspension. Next, 0.1 g of sodium metabisulfite was added to the suspension and stirred at 85 °C for 120 min to break the secondary and quaternary structures of the SPI to obtain a stable SPI hydrosol. Finally, 0.5 g of SDS was added to the hydrosol to prevent demulsification during the graft copolymerization.

#### 2.2.2. Waterborne Polyacrylate Synthesis

An alkaline aqueous solution (100 mL) that contained 0.4 wt·% NaOH was prepared. MMA, BA, and AAEM were washed with the alkaline aqueous solution three times to remove the inhibitor of the acrylic monomer. The monomer was washed with 100 mL of distilled water three times to remove the alkaline aqueous solution from the monomer.

An ammonium persulfate initiator (0.042 g) was dissolved in 15 g of distilled water. SDS (0.60 g) and OP-10 (0.30 g) were dissolved in 45 g of distilled water to form an emulsifier solution for later use. The MMA (18 g), AAEM (5.9 g), and BA (15 g) were mixed for later use.

The emulsifier solution was added into a four-necked flask, and the water bath temperature was adjusted to 80 °C. After the flask was filled with nitrogen, 10 wt·% of the monomer and 10 wt·% of the initiator were poured into the four-necked flask and mechanically stirred at 360 rpm for pre-emulsification for 15 min. The monomer and initiator were added dropwise to the four-necked flask at rates of 1 drop/3 s and 1 drop/30 s, respectively.

#### 2.2.3. Preparation of SGA Emulsion

After the monomers were dripped slowly to completion, the modified SPI hydrosols with 1 g, 3 g, 4 g, and 6 g SPI were added directly to the flask containing the polyacrylate emulsion. The samples were named A2, A3, A4, and A5, respectively. The unreacted monomer and free radical in the emulsion were used for graft copolymerization. The reaction time of the graft copolymerization was 2 h. The pure polyacrylate emulsion was named A_1_. When the amount of soy protein was over 6 g, the emulsion was unstable, and demulsification occurred.

#### 2.2.4. The Measurement of Grafting Ratio of SGA

After completion of the reaction, the flask was cooled under running tap water and the final product was precipitated with an ethanol solution for 24 h. The rudimentary product was then extracted with acetone for 48 h to remove the homopolymer and any unreacted monomer, dried at 40 ℃ for 12 h, and subsequently at 70 ℃ for 48 h to remove water. The percentage of grafting was calculated using Equation (1). The grafting ratio of SGA with different SPI content is shown in Table 1.

(1) $$GP\left(\%\right)=\frac{W_{1}-W_{0}}{W_{0}}\times 100\%$$

where W_0_ and W_1_ denote the weights of the SPI and SGA, respectively.

#### 2.2.5. Preparation of Film and Coating

Polyacrylate film preparation: The coatings were separately placed in a Teflon plate, and all the samples were placed in a 50 °C infrared oven for 4 h to remove moisture before use.

Wooden coating preparation: The ash veneer (30 cm × 20 cm × 12 cm) was smoothed in the grain direction. After the swarf was removed, the self-synthesized water-based acrylate emulsion was evenly applied to the surface of the veneer and cured at 40–50 °C for 2 h. The above operation was repeated two times. We tested the performance of the paint film after it was completely dry after 10 days.

#### 2.2.6. Tests and Characterization

The surface functional groups of the SPI, acrylic resin, and SGA copolymer were characterized using Fourier-transform infrared spectroscopy (FTIR, MagnaIR500 E.S.P, Nicolet Company, Tramelan, Switzerland) from 400 to 4000 cm^−1^ with a resolution of 4 cm^−1^ in reflection mode. Scans were conducted 40 times/min. The final spectra were obtained by averaging 3 scans. The morphologies of the SPI and SGA were observed using transmission electron microscopy (TEM, JEM-2100, Japan Electronics Corporation, Tokyo, Japan). A small amount of the emulsion was diluted with distilled water and stained with a 2 wt·% phosphotungstic acid solution. The particle size distributions of the emulsions were characterized using a ZetaPALSNanoDLS (Brookhaven Co., New York, NY, USA). The emulsions were diluted to about 1 wt·% with deionized water. The test was carried out using a dynamic mechanical analyzer (DMA) in tensile mode with a sample size of 15 mm × 0.25 mm × 6.5 mm, a heating rate of 5 K/min, a frequency of 1 Hz, an amplitude of 20 μm, and a temperature range of −50 to 150 °C. The mechanical properties of the films were evaluated using a universal mechanical testing machine (UTM2203). The elastic modulus, tensile strength, and elongation at break of the film before and after modification were measured at a tensile speed of 5 mm/min. Wood-coating performance tests, including abrasion resistance, hardness, adhesion, and gloss tests, were performed according to the GBT4893.8–2013, GB/T 6739-2006, GBT4893.4–2013, and GBT4893.6–2013 standards, respectively.

## 3. Results and Discussion

### 3.1. FTIR Analysis of Polyacrylate and SGA

**Figure 2:**
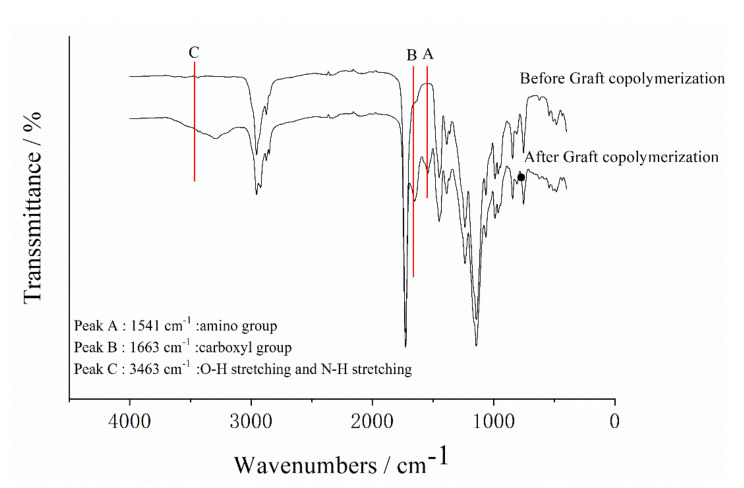
FTIR spectra of polyacrylate and SGA.

FTIR spectra of the polyacrylate and SGA are shown in Table 2 and Figure 2. According to Table 1, the strong absorption band at 3463 cm^−1^ was generated by the stretching vibrations of the O–H and N–H groups [34]. Furthermore, the characteristic amide I band at 1663 cm^−1^ (C=O stretching vibration) and amide II at 1541 cm^−1^ (N–H bending and C–N stretching vibrations) were observed in SGA spectrum [36,37]. The important absorption bands for polyacrylate were at 1736 cm^−1^ and 1180 cm^−1^. They were caused by C=O stretching vibrations and C–O stretching vibrations, respectively. Compared with the spectra of polyacrylate and SGA, the observed absorption bands for the graft copolymerization indicated that the SPI had grafted onto polyacrylate.

### 3.2. TEM of Polyacrylic Emulsion and SGA

**Figure 3:**
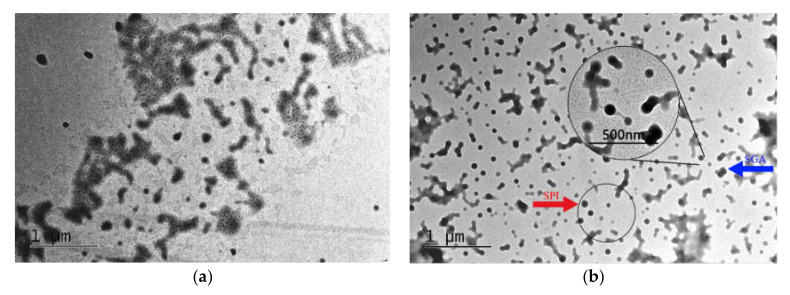
TEM image of the polyacrylic emulsion and SGA: (**a**) polyacrylate, (**b**) graft copolymer SGA.

The polyacrylic emulsions obtained before and after graft copolymerization were observed using TEM. 7S globulin and 11S globulin are the dominant proteins in SPI. In TEM, SPI micelles are solid and spherical in shape [40]. As shown in Figure 3a, before the graft copolymerization, the latex particle diameter of the polyacrylic resin emulsion was about 60 nm, and the emulsion particles were irregular shapes. The TEM image of the SGA graft copolymer emulsion is shown in Figure 3b. The spherical single dots with clear boundaries were considered as SPI, as highlighted by the red arrow in Figure 3b. The blue arrow in the figure points to the graft copolymer of the SGA. The amino and carboxyl groups on the SPI were attacked by the initiator, ammonium persulfate (APS), to form free radicals, which then reacted with acrylate monomers. The enlarged region in Figure 3b further confirmed that the SPI had been successfully grafted onto the polyacrylate.

### 3.3. Analysis of Particle Size and Distribution of Emulsion

**Figure 4:**
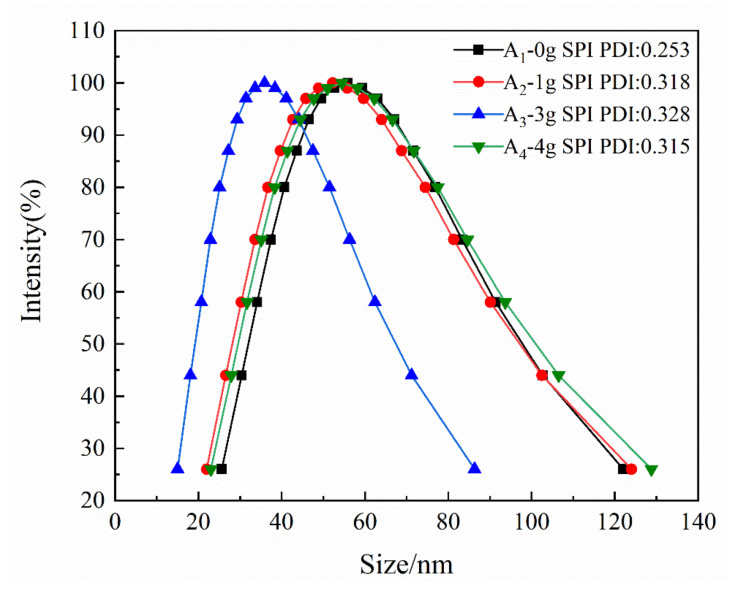
Particle size distribution curve of emulsion.

The particle-size distributions and polydispersity indices of emulsions A1–A4 are shown in Figure 4. As the reaction proceeded, the particle size increased, and the emulsion changed from light blue to opaque white. The average diameter of the emulsion particles was between 35 and 56 nm. The polydispersity index (PDI) ranged from 0.253 to 0.328, which corresponded to a narrower particle size distribution and good stability with no layering and caking after two months.

### 3.4. Dynamic Mechanical Analysis

**Figure 5:**
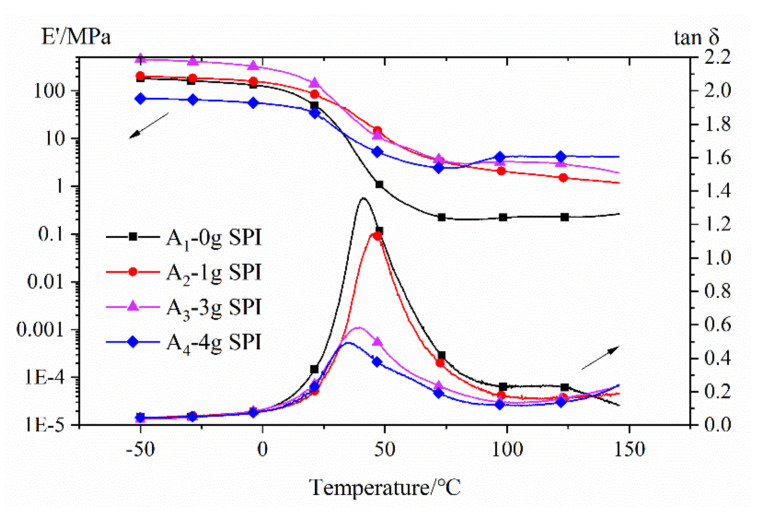
Storage modulus (E’) and the damping coefficient (tanδ) of the films. (Note: the film fabricated with 6 g of SPI was too brittle to be tested using dynamical mechanical analysis (DMA)).

The storage moduli (E’) and the damping coefficients (tanδ) of the films are shown in Figure 5. The dynamical mechanical analysis (DMA) curves for these samples were obtained from rectangularly shaped specimens subjected to a heating cycle at a rate of 3 °C min^−1^ and a frequency of 1 Hz. As shown in Figure 5, the storage modulus of the solidified membrane in the glass transition state increased first and then decreased as the soy protein isolate content increased. This increase in the storage modulus was attributed to the successful grafting of acrylic resin onto the SPI, which resulted in covalent bond formation between the two reaction species. When the content of soybean protein was 4 g, more hydrophilic NH_2_/OH groups were exposed. The lower storage modulus was attributed to the inhomogeneity caused by a hydrophobic polymer matrix and hydrophilic NH_2_/OH groups in the emulsion [38,41]. The glass transition temperature (Tg) increased first and then decreased as the soy protein isolate content increased. In general, the peak intensity of Tg in the tanδ curve was inversely proportional to the volume fraction of the confined polymer segments in the bulk polymer [42,43]. As the amount of added soy protein increased, the graft density increased, and the grafted chains grew longer. As a result, the spacing between the chains and the free volume increased. The easier the molecular motion was, the lower the value of Tg became.

The storage modulus E’ is a measurement of a material’s stiffness and can be used to provide information regarding the crosslink density. The crosslink density (ν_e_) can be calculated using the following formula:

ν_e_ = E’/3RT (2)

where E’ is the storage modulus of the thermoset in the rubbery plateau region at Tg + 40 °C, R is the gas constant, and T is the absolute temperature. The calculated results are shown in Table 3.

### 3.5. Analysis of Film Mechanical Properties

**Figure 6:**
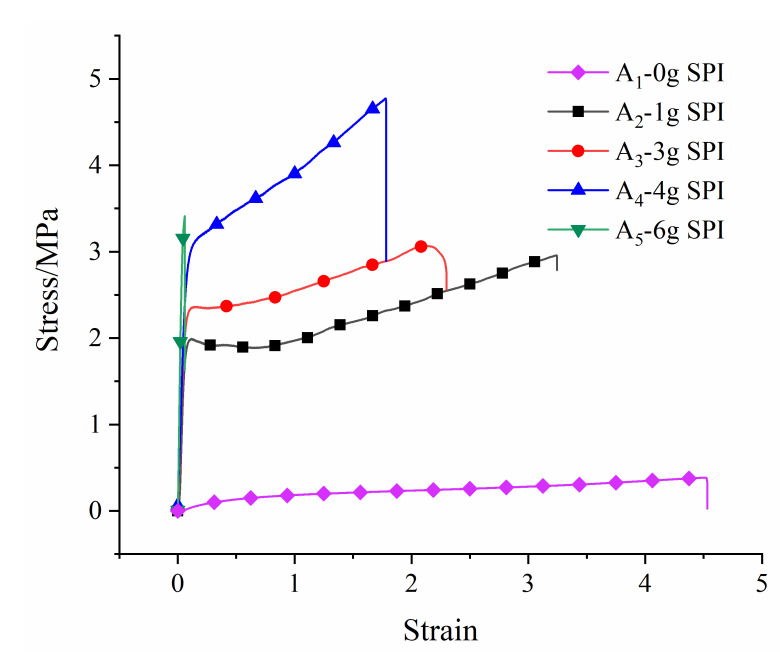
Stress–strain curves of copolymers.

The stress–strain curves of the copolymer and mechanical properties are shown in Figure 6. With the increase in the SPI content, the strain decreased, and the elastic modulus increased. Table 4 shows the data for the tensile tests of the films. The results show that as the soy protein fraction in copolymer increased from 0% to 11.38%, the Young’s modulus of the film increased, the elongation at break decreased, and the tensile strength increased first and then decreased. When the soy protein content reached 11.38%, the tensile strength reached 102.343 MPa, but the elongation at break was only 21.416%, and the film was brittle. When the soy protein content reached 7.28%, the elastic modulus, tensile strength, and elongation at break were 46.949 MPa, 4.775 MPa, and 187.125%, respectively, which had better toughness. In general, the soy protein content had a significant effect on the mechanical properties of the film. Furthermore, the wear resistance and scratch resistance of the coating were also improved.

### 3.6. Performance Analysis of Wood Coating

The wear resistance, adhesion, gloss, and pencil hardness of the paint film were tested using the paint film, a grinder, a gloss meter, and a pencil hardness tester, respectively. The results are shown in Table 5. The adhesion grade decreased after the SPI grafting. However, the wear resistance, gloss, and pencil hardness of the composite material improved. The glossiness of the paint film (60°) increased first and then decreased with the increase in the amount of SPI grafting. The highest gloss was obtained for the A4 group. However, it did not exceed 70, and thus, it was in the semi-gloss range. The hardness increase was the most significant, and the A4 and A5 pencil hardness reached 3H. However, A5 was more brittle, and thus, A4 was superior when all the properties were considered together.

## 4. Conclusions

Soy protein isolate was introduced to modify an acrylic emulsion to address the unsatisfactory mechanical behavior and hardness. O–H, N–H groups, amino groups, and carboxyl groups appeared in the FTIR spectra of the SGA due to the SPI. TEM analysis further confirmed that SPI was successfully grafted onto the polyacrylic resin. Compared with polyacrylic acid resin, the Tg of the SGA graft copolymer decreased, and the maximum degree of crosslinking reached 0.35 × 10^3^ mol m^−3^. With the increase in the SPI content, the tensile strength and elastic modulus increased gradually, whereas the elongation at break decreased gradually. When the SPI addition reached 4 g, the film exhibited desirable properties: the elastic modulus, tensile strength, and elongation at break were 46.949 MPa, 4.775 MPa, and 187.125%, respectively. The abrasion loss after 100 revolutions, adhesion level, gloss, and pencil hardness were 0.022 g, 1, 67.3, and 3H, respectively.

The modification of polyacrylic resin by SPI graft copolymerization thus provides a method for the biomass modification of wood coatings.

## Figures and Tables

**Table 1:** Grafting ratio of soy protein isolate–grafted–acrylate (SGA) with different SPI content.

Group	A1	A2	A3	A4	A5
$\mathrm{GP}\left(\%\right)$	/	43%	40%	45%	49%

**Table 2:** FTIR spectra of the polyacrylate and SGA.

FTIR Band	Attribution Group
3463 cm^−1^	stretching vibrations of the O-H and N-H groups (SPI)
1736 cm^−1^	C=O stretching of the ester carbonyl (polyacrylate)
1663 cm^−1^	amide I (C=O stretching vibration) (SPI)
1541 cm^−1^	amide II(N–H bending and C–N stretching vibrations) (SPI)
1460 cm^−1^	methyl and methylene groups(polyacrylate)
1152 cm^−1^	C-O stretching of the corresponding ester (polyacrylate)

**Table 3:** Crosslinking density of films with different SPI content.

	0 g SPI	1 g SPI	3 g SPI	4g SPI
Tg (°C) (tanδ max from DMA)	41.4	45.4	38.8	35.0
Storage modulus (E’) (MPa) at 25 °C (glassy state)	38.12	68.59	105.99	25.91
Storage modulus (E’) (MPa) at Tg + 40 °C (rubbery state)	0.20	2.52	3.09	2.41
ν_e_ (× 10^3^ mol m^−3^)	0.02	0.28	0.35	0.28

**Table 4:** Mechanical properties of copolymers with different SPI mass fractions.

Group	Soy Protein Fraction in Copolymer (%)	Elastic Modulus (MPa)	Elongation at Break (%)	Tensile Strength (MPa)
A_1_	0	0.3170	453.133	0.385
A_2_	1.72	33.515	335.137	2.955
A_3_	5.75	41.558	240.353	3.064
A_4_	7.28	46.949	187.125	4.775
A_5_	11.38	102.343	21.416	3.045

**Table 5:** Wear resistance, adhesion, gloss, and pencil hardness of the paint film with different SPI additions.

Group	Abrasion Loss of 100 Revolutions (g)	Adhesion (Level)	Gloss 60° (Value)	Pencil Hardness
**A_1_**	0.032	0	48.2	HB
**A_2_**	0.015	0	55.1	HB
**A_3_**	0.023	1	65.1	2H
**A_4_**	0.022	1	67.3	3H
**A_5_**	0.020	2	66.7	3H
